# The Effect of University Students’ Emotional Intelligence, Learning Motivation and Self-Efficacy on Their Academic Achievement—Online English Courses

**DOI:** 10.3389/fpsyg.2022.818929

**Published:** 2022-02-16

**Authors:** Yuan-Cheng Chang, Yu-Ting Tsai

**Affiliations:** ^1^Department of Education Management, Chinese International College, Dhurakij Pundit University, Bangkok, Thailand; ^2^Department of International Business, Chinese International College, Dhurakij Pundit University, Bangkok, Thailand

**Keywords:** emotional intelligence, learning motivation, self-efficacy, academic achievement, pandemic (COVID-19)

## Abstract

The COVID-19 pandemic has had a significant impact on education worldwide. The disease first hit China and numerous Chinese cities then started to conduct online courses. Therefore, this study aims to explore the effect of the Shanghai students’ emotional intelligence, learning motivation, and self-efficacy on their academic achievement when they participated in online English classes during the latter phase of the pandemic in China. Furthermore, the research also examines whether the students’ emotional intelligence can influence their academic achievement through the mediation effect of their learning motivation and self-efficacy. Social Cognitive Theory (SCT) and the social cognitive Expectancy-Value Model were employed to build the research framework, and the method of structural equation modeling (SEM) was utilized to conduct the model verification. Ten universities in Shanghai, China were selected for sampling. In total, 450 students were surveyed of which 404 questionnaires were valid. The results show that the students’ emotional intelligence did not directly affect their academic achievement. Nevertheless, the students’ emotional intelligence had a positive effect on their learning motivation and self-efficacy. In addition, mediation analysis showed that the relation between emotional intelligence and academic achievement was sequentially mediated by learning motivation and self-efficacy.

## Introduction

The COVID-19 pandemic has had a significant impact on education. There have been several schools closed in 180 countries or regions since the end of April 2020 and 85% of students could not go to school (World Bank, 2020a,b). The COVID-19 pandemic has been a typically adaptive and revolutionary challenge for educators, who needed to take countermeasures rapidly. Thus, numerous schools worldwide have managed to continue to teach online with their resources during the pandemic (Reimers et al., 2020).

There are several factors influencing students’ online academic achievement. A body of recent studies have shown that emotional intelligence (EI) (Berenson et al., 2008), learning motivation (Nonis and Fenner, 2012), and self-efficacy (Cussó-Calabuig et al., 2018; Yokoyama, 2019) have an effect on academic achievement.

Mortiboys (2012) points out that there have been various scholars interested in the effect of EI on education and there has been a dramatic increase in the number of studies on that (Perera, 2016). Mayer et al. (2008) suggested that EI refers to how people manage, comprehend, and use their relevant emotional traits and cognitive ability when they get along with others. EI also means that individuals’ social intelligence enables them to recognize and differentiate their own and others’ emotions in order to make appropriate decisions and take responsive actions (Alhebaishi, 2019). In terms of language learning in EI, emotional characteristics and cognitive ability are beneficial to reading comprehension (Motallebzadeh, 2009; Abdolrezapour and Tavakoli, 2012), introspection (Afshar and Rahimi, 2016; Chang, 2021), speaking (Asadollahfam et al., 2012), listening comprehension (Serraj, 2013), and writing performance (Pishghadam, 2009; Shao et al., 2013). Moreover, high EI has a positive impact on language development (Rostampour and Niroomand, 2013; Kourakou, 2018) and language learning strategies (Aghasafari, 2006).

Dubey (2012) found that students’ EI was positively correlated with their learning motivation. Henter (2014) also proposed that EI, motivation, and linguistic performance correlated positively. According to Schunk and Meece (2005), motivation is a deep mental phenomenon, normally defined as the strength of dominating individuals’ behavior, and drives them to be engaged in goal-directed behavior (Jenkins and Demaray, 2015). Furthermore, Bain et al. (2010) pointed out that students’ motivation was connected to the effectiveness of their learning. Students’ learning could also be maintained through the stimulation of motivation. Tella (2007) reported that it was difficult to reach satisfactory learning outcomes if there was a lack of learning motivation. Ivanova et al. (2019) noted in their research of second language learning that students’ learning motivation influenced their grades of foreign languages. As a result, learning motivation was essential since it was closely related to academic achievement and performance (Titrek et al., 2018; Duchatelet and Donche, 2019).

Self-efficacy plays a vital role in learning processes and learning outcomes (Zhang and Ardasheva, 2019). It allows learners to be more involved in their learning processes regarding their motivation, cognition and behavior (Anam and Stracke, 2016). One of the components of social cognition is self-efficacy; Bandura defined self-efficacy as one’s belief in his or her ability to achieve assignments (Bandura, 2001). The major element of personal efficacy in mankind’s accomplishments, attitude, and performance is belief, which is an important component in Social Cognitive Theory (SCT) (Kirk et al., 2008). In addition, Morali (2019) suggested that reading self-efficacy and attitude has a crucial predictive effect on EFL (English as a foreign language) reading comprehension achievement (Rachmajanti and Musthofiyah, 2017).

Bandura (1997) connected the function of efficacy and the concept of EI in his research and considered that the control of self-awareness and emotions might be linked with higher levels of self-efficacy. Gundlach et al. (2003) also indicated that EI could influence self-efficacy through emotions and the process of causal reasoning, which impacted important work outcomes. Moreover, students’ self-efficacy had the mediation effect between EI and academic performance (Udayar et al., 2020). Therefore, students’ emotional intelligence and the ability to manage their emotions affect both their learning motivation and belief in their ability and performance. Furthermore, students’ EI is helpful for enhancing their learning results owing to the belief in their own ability (Udayar et al., 2020).

As mentioned above, students’ EI exercises an influence on their learning motivation, self-efficacy, and academic achievement. Additionally, students’ learning motivation and self-efficacy impact their academic achievement. Under the effect of the COVID-19 pandemic, most schools have been utilizing online teaching (Reimers et al., 2020). However, online teaching is distinct from traditional methods. Teachers, students as well as classmates can not discuss face-to-face, which may lead to different learning outcomes, as students’ emotional cognition, the control of their emotions, and the way they express themselves online may be dissimilar from those offline. Consequently, the major purpose of this study is to explore the relationship among university students’ EI, learning motivation, self-efficacy, and English academic achievement when they take online English courses. The research is based on SCT and the social cognitive Expectancy-Value Model (E-VM) of achievement motivation.

In this paper, a model is built to discuss the relationship among university students’ EI, learning motivation, self-efficacy, and English academic achievement. Moreover, in order to verify the model, structural equation modeling (SEM) is applied to it. The aim of this research is threefold:

1. to explore the effect of university students’ emotional intelligence on their learning motivation, self-efficacy, and academic achievement when they take online courses.
2. to explore the mediation effect of university students’ self-efficacy between their learning motivation and academic achievement when they take online courses.
3. to explore whether university students’ emotional intelligence has an indirect effect on their academic achievement through their learning motivation and self-efficacy when they take online courses.

### Emotional Intelligence and Academic Achievement

The concept of EI was proposed by Salovey and Mayer earliest (Salovey and Mayer, 1990; Mayer and Salovey, 1993; Bar-On, 1997). According to their research, EI was defined as individuals’ ability to monitor and discriminate their own and others’ feelings and emotions, which could guide their thoughts and behavior. Furthermore, EI is a set of cognitive abilities and emotional competencies, which are connected (Ciarrochi et al., 2001). It also refers to the ability that lets people differentiate, express, control, and utilize their emotions through self-adaptive approaches (Nordin, 2012; Shafiq and Rana, 2016). Humans need to sense their own and others’ feelings to enable themselves to adapt to social behavior (Salovey and Mayer, 1990; Mayer and Salovey, 1993). Emotion perception includes how people alter their own emotions and modify them towards others, and what emotional content they utilize when resolving problems (Salovey and Mayer, 1990; Mayer and Salovey, 1993). EI is a tendency where individuals are likely to distinguish, evaluate and cope with their own and others emotional states in order to achieve particular goals (Fox and Spector, 2000; Choudary, 2010). Mayer et al. (2000) considered that EI was a zeitgeist, which comprised a group of personality traits and a set of abilities that processed related emotional information. The term zeitgeist also implied the combination of individuals’ emotions and rationality in human history (Mayer et al., 2000).

The cognitive structure of EI consisted of the following four parts: “emotional self-assessment,” “self-expression assessment,” “identification of others’ emotions for emotional self-regulation,” and “the use of emotions to facilitate performance” (Mohammad et al., 2009). Emotions make people’s cognitive processes adjustable and let them have rational thinking (Brackett et al., 2011) and EI allows individuals to have the ability to appreciate and discriminate emotions (Prati et al., 2003). In other words, EI empowers individuals to know how to merge their rationality and emotions (Mayer et al., 2000). Hence, EI refers to one’s acceptance of emotions and his or her use of those in order to make appropriate decisions in life and interpersonal relationships (Karimi et al., 2014; Vidyarthi et al., 2014). It also refers to the understanding of ourselves and others, the self-control of immediate requirements, peoples’ empathy, and the positive exercise of emotions (Karimi et al., 2014; Vidyarthi et al., 2014). Furthermore, Goleman et al. (2013) proposed that EI encompasses individuals’ ability to manage their emotions effectively and their capacity to master their emotions and impulses when they feel like a failure, depressed, and disappointed. They also stated that EI is people’s competence in constraining their feelings in interpersonal relationships and encouraging or guiding others when they get on with each other.

In order to create effective learning opportunities in the educational environment, students not only need to gain knowledge at school, but also to cultivate social and emotional abilities (Amirian and Behshad, 2016). Numerous studies have noted that EI is pertinent to success in several fields including effective teaching (Ghanizadeh and Moafian, 2009), students’ learning (Brackett and Mayer, 2003), and academic achievement (Márquez et al., 2006; Fallahzadeh, 2011). In addition, EI, academic achievement and other emotional and cognitive characteristics, which were helpful for learning, were proven positively correlated through empirical research. In the research of Shamradloo (2004), EI could predict one’s academic achievement twice as much as cognitive intelligence. As a consequence, the study of students’ emotional intelligence is beneficial for facilitating their academic achievement. The first research hypothesis is as follows:

**H_1_**: Emotional intelligence has a positive effect on academic achievement.

### Emotional Intelligence and Self-Efficacy

Self-efficacy was a crucial individual variable from Bandura’ SCT (Bandura, 1986), which emphasized the significance of social experience and the necessity of observational learning in the process of developing character (Mahler et al., 2018). Bandura (1997) also defined self-efficacy as individuals’ belief in their own competence in arranging and carrying out operations to create the expected accomplishments and outcomes. In Qureshi’ investigation, the interaction of cognition (personal factor), behavioral element and environmental component determined one’s behavior (Qureshi, 2015). To put it in another way, individuals’ decisions in certain situations depended on their own observation. The observation of others’ behavior in one’s memory would influence his or her cognitive process and social behavior in future events. Bandura (1994) suggested that individuals with high self-efficacy had various positive traits that are comprised of having confidence in one’s ability to handle arduous tasks and then continuing to work on them. Other characteristics include setting challenging objectives and then proceeding with them, putting more effort into assignments and then reviving positive self-efficacy after experiencing failure and encountering obstacles (Bandura, 1994). Self-efficacy enables us to control our thoughts, feelings, and behaviors; it is also concerned with people’s belief in their competence (Baron et al., 2016; Halper and Vancouver, 2016). Self-efficacy involves individuals’ perspective on what they can and cannot do (Bandura, 1997; Kirk et al., 2008). The belief in self-efficacy, which was a key element in SCT, played a vital role in mankind’s accomplishments, attitudes, and performance (Bandura, 1997; Kirk et al., 2008). On the contrary, people with low self-belief or low self-efficacy might suppose that things were more strenuous than reality, which contributed to the increase in pressure as well as depression, and tunnel vision in problem-solving (Pajares and Schunk, 2001).

With respect to the relation between EI and self-efficacy, Salovey and Mayer (1990) showed that the concept of EI was individuals’ ability to deal with their emotions. They also defined EI as the competence in monitoring and distinguishing emotions, which were applied to leadership mindset and behavior. Moreover, managing this kind of self-awareness was essential to the adjustment of emotions (Bandura, 1997). Self-awareness was tied closely with self-efficacy, since self-efficacy gave prominence to self-awareness and self-regulation (Bandura, 1997). This element affects the development of self-efficacy.

Bandura (1997) observed that when people recognized thoughts, feelings and behavior to explain organizational reality through their self-awareness, self-regulation and self-control, their EI and self-efficacy would be internalized (Bandura, 1997).

The emphasis on self-awareness, self-regulation and self-control was the major component causing the development and realization of self-efficacy in SCT, which was similar to the area of research that was focused on in the study of EI (Gundlach et al., 2003). From this point of view, some researchers have considered that the studies on self-efficacy and EI are interrelated. The main reason for that is EI can assist individuals to produce the causal attributions that damage their belief in self-efficacy the least, through altering their possible emotions (Gundlach et al., 2003). Furthemore, Emmer and Hickman (1991) suggested that researchers could explore the relationship between emotions and the belief in efficacy in academic settings.

In groundbreaking study Bandura’s (1997), the effect of efficacy and the framework of EI were linked. He considered that the control of self-awareness and emotions might result in higher degrees of self-efficacy.

There have been several studies showing that EI and self-efficacy are closely connected and positively correlated (Kirk et al., 2008; Rastegar and Memarpour, 2009; Hamdy et al., 2014; Gurbuz et al., 2016). It may be difficult for people with low EI and self-efficacy to complete their daily tasks in order (Rostami et al., 2010). Furthermore, serious anxiety contributes to the decrease in performance, which then reduces self-efficacy. As a result, individuals with high EI can manage their emotions and actively handle problems.

Emotional intelligence influences one’s ability to control his or her self-efficacy through causal reasoning and it also impacts essential work results (Gundlach et al., 2003). Chan (2007) and Mikolajczak and Luminet (2007) also found that people who appeared to have high EI had higher self-efficacy. Nonetheless, more investigation needs to be conducted to explore which elements of EI play a more significant role in demonstrating the changes in self-efficacy (Shipley et al., 2010). In SCT, the ability to control emotions and self-efficacy are related (Bandura, 1997; Gundlach et al., 2003), and emotional intelligence affects self-efficacy (Mikolajczak and Luminet, 2007; Hamdy et al., 2014; Gurbuz et al., 2016). As has been discussed, the second research hypothesis is as follows:

**H_2_**: Students’ emotional intelligence has a positive effect on their self-efficacy

### The Relationship Among Emotional Intelligence, Learning Motivation, Self*-*Efficacy, and Academic Achievement

Motivation is the ability in which individuals encourage themselves and others to conduct a certain behavior or a series of behaviors; it also enables people to achieve great accomplishments (Rahim and Psenicka, 2002). Keller (1987) introduced the ARCS model (ARCS stands for attention, relevance, confidence, and satisfaction) to seek a more constructive approach to comprehend what greatly influences motivation and search for a systematic method to recognize and resolve problems concerning learning motivation. Doménech-Betoret et al. (2017) considered that one of the most reliable approaches to linking variables such as learning motivation, self-efficacy and academic achievement was employing the social cognitive E-VM (Eccles, 1983; Wigfield and Eccles, 1992, Wigfield and Eccles, 2000). This model encompasses a variety of components and connections that are divided into three blocks or categories of variables, and these are “social world”, “cognitive processes” and “motivational beliefs” in sequence. All of the blocks of variables can be directly or indirectly utilized as a predictive index of students’ willpower, options and achievement behavior. This model brought up a hypothesis based on motivational beliefs. First, people’s expectations of success and subjective task values are directly associated with accomplishments, options of assignments and determination. Second, “expectancies and task values” are affected by people’s objectives and “self-schemata.”

Moreover, self-efficacy and individuals’ beliefs in their own ability can be viewed as a significant part of self-schemata. Elliot (1999) defined achievement motivation as the route of competence-based affect, cognition, and behavior which stimulated the course of accomplishment leading students to failure or success. The crucial evidence, provided by past research on verified structural models based on the expectancy value theory, approves of the fact that the variables of motivational expectancy value play an essential role in students’ self-beliefs (such as self-efficacy, self-concept, and self-esteem) and academic achievement (Doménech-Betoret et al., 2014, 2017). It also emphasizes the significance of the variables of motivational expectancy value in terms of their prediction of students’ academic achievement.

**H_3_**: Self-efficacy has the mediation effect between learning motivation and academic achievement

Therefore, SCT and the social cognitive EV-M can be utilized to explain the relationship among EI, learning motivation, self-efficacy and academic achievement. Dubey (2012) found a positive correlation between EI and learning motivation; moreover, students with high, medium and low levels of motivation had a significant difference in EI. Additionally, Henter (2014) reported that EI could enhance motivation and linguistic performance, and it had a positive impact on self-efficacy (Ngui and Lay, 2020). Individuals with high EI could also accommodate themselves to different types of lifestyles, make use of effective coping skills when encountering problems and have self-efficacy (Shipley et al., 2010). Gharetepeh et al. (2015) showed that EI correlated positively with self-efficacy and could be used to forecast academic achievement, and self-efficacy was a major factor in successful performance (Baron et al., 2016). Usher and Pajares (2008) also pointed out that self-efficacy could predict student academic achievement in every academic area. Students’ self-efficacy, sense of responsibility for their projects and GPAs of their final exams were positively correlated (Zimmerman and Kitsantas, 2005; Yazici et al., 2011). Doménech-Betoret et al. (2017) also notes that there have been a considerable body of studies showing that the belief in self-efficacy directly influences academic achievement. Consequently, students’ ability to control their emotions affects the creation of their learning motivation, which also impacts self-efficacy and eventually influences academic achievement.

**H_4_**: Emotional intelligence has a positive effect on learning motivation.

**H_5_**: Learning motivation and self-efficacy have the mediation effect between emotional intelligence and academic achievement.

## Materials and Methods

### Participants

There have been a considerable number of universities in China utilizing online teaching due to the COVID-19 outbreak. Shanghai is one of the first-tier cities in China and is better equipped with educational facilities. Thus, the participants in this study were university students in Shanghai, China, majoring in Business Management. One hundred and fifty students were selected from three universities for pre-testing. Ten universities running online English courses were selected through purposive sampling, with one class drawn from each of the universities, and 45 students drawn from each class. The questionnaires were distributed by the students’ teachers and they filled them out online. In total, 450 students were surveyed and 432 questionnaires were retrieved. With invalid questionnaires excluded, a total of 404 valid questionnaires were captured. 149 of the respondents were male and 255 were female.

### Instruments

The students’ academic achievement was measured by their scores ranging from zero to 100 of an English final examination. The average score of the participants was 80.978. The maximum was 100, and the minimum was 24. The standard deviation was 11.819.

The ARCS Model’s four constructs (Attention, Relevance, Confidence, and Satisfaction) proposed by Keller (1987) were employed to design the survey questions for the Chinese students’ learning motivation, which includes 10 questions with scaled responses, for example “The course’s teaching style motivates me to actively learn.”, “This course is very interesting.”, “I think the content of this course is worth learning.”

In terms of the reliability analysis of the pre-testing scale, the Cronbach’s alpha was 0.931, which showed good reliability. Moreover, confirmatory factor analysis (CFA) was conducted to test the returned questionnaires. The factor loading for all questions in the survey recorded between 0.648 and 0.837. The construct reliability (CR) value of the scale was 0.932, exceeding the evaluative criteria of 0.60. The average variance extracted (AVE) value of the scale was 0.579, exceeding the evaluative criteria of 0.50 (Fornell and Larcker, 1981). This indicates that the scale had a high level of construct validity and discrimination. As for the scale’s goodness of fit test, the results were as follows: SRMR = 0.048, χ^2^/*df* = 6.099, GFI = 0.899, AGFI = 0.841, PGFI = 0.572, NFI = 0.920, IFI = 0.932, CFI = 0.932, PNFI = 0.716, RMSEA = 0.112, which shows that the scale had a satisfactory goodness of fit.

The self-efficacy scale, comprising of 10 questions, proposed by Scholz et al. (2002), was adopted for estimating self-efficacy. The research subjects were Chinese students; therefore, the questionnaire was translated into Mandarin by a translator. In order to verify the accuracy of the translation, the Mandarin version of the survey was then translated back into English by another translator. The reliability analysis shows that the Cronbach’s alpha was 0.891. In terms of CFA, the factor loadings of all questions recorded between 0.595 and 0.813, with a CR of 0.892 and an AVE of 0.457, which indicates that the reliability and credibility of the scale were still acceptable. The results were as follows: SRMR = 0.048, χ^2^/*df* = 4.797, GFI = 0.923, AGFI = 0.879, PGFI = 0.588, NFI = 0.906, IFI = 0.924, CFI = 0.924, PNFI = 0.705, RMSEA = 0.097, which shows that the scale had a satisfactory goodness of fit.

The Wong and Law Emotional Intelligence Scale consists of four dimensions including self-emotion appraisal (SEA), others’ emotional appraisal (OEA), use of emotion (UOE), and regulation of emotion (ROE) (Wong and Law, 2002). This was the scale employed to design the survey questions. Each of the above mentioned aspects comprised of four questions and (16 questions in total). The questionnaire was also translated into Mandarin by a translator and translated back to verify accuracy. In terms of the reliability analysis of the scale, the Cronbach’s alpha was 0.929. In terms of CFA, the factor loadings of SEA recorded between 0.626 and 0.878, with a CR of 0.860 and an AVE of 0.610. The factor loadings of OEA recorded between 0.796 and 0.856, with a CR of 0.899 and an AVE of 0.691. The factor loadings of UOE recorded between 0.626 and 0.818, with a CR of 0.841 and an AVE of 0.573. The factor loadings of ROE recorded between 0.821 and 0.858, with a CR of 0.906 and an AVE of 0.707. The results were as follows: SRMR = 0.048, χ^2^/*df* = 3.046, GFI = 0.922, AGFI = 0.892, PGFI = 0.665, NFI = 0.937, IFI = 0.956, CFI = 0.956, PNFI = 0.781, RMSEA = 0.071.

## Results

In terms of research results, the data were tested first for serious common method variance (CMV), then for differential validity and correlation analysis, and finally for overall path model analysis.

### Common Method Variance

This study used Harman’s single-factor test to examine the CMV (Aulakh and Geneturk, 2000). The first part consisting of five factors extracted with the exploratory factor analysis (EFA) account for 43.051% of the total variance, which is less than 50%, indicating that the common method variance was not of great concern (Aulakh and Geneturk, 2000; Podsakoff et al., 2003).

Next, the confirmatory factor analysis (CFA) was adopted to compare the single-factor and multi-factor models. The single-factor model constitutes a one-factor structure for all dimensions, whereas the multi-factor model has a fully correlated structure for the theoretical CFA. The single-factor and multi-factor models were compared to observe if any significant difference existed in their overall levels of goodness-of-fit, degrees of freedom, and chi-square values. A significant difference would indicate that the multi-factor model achieved a higher level of goodness-of-fit than the single-factor model, and that the single-factor structure was not present; therefore, the CMV was not serious (Mossholder et al., 1998; Iverson and Maguire, 2000). As can be seen in Table 1, the multi-factor model performed better than the single-factor model in all indicators for the overall level of goodness-of-fit (χ^2^/DF, GFI, AGFI, NFI, CFI, SRMR), and the comparison of the degrees of freedom and chi-squared values between the two models displayed significant differences (Δχ^2^= 2,441.377, ΔDF = 15, *p* = 0.000). On this basis, this study does not have serious common method variance.

**TABLE 1:** Difference between single-factor model and multi-factor model.

Model	χ^2^	DF	Δχ^2^	ΔDF	*p*	χ^2^/DF	GFI	AGFI	NFI	CFI	SRMR
Single factor model	3,816.342	594	2,441.377	15	0.000	6.42	0.522	0.464	0.633	0.670	0.090
Multi-factor model	1,374.965	579				2.375	0.835	0.810	0.868	0.918	0.051

### Discriminant Validity and Relevant Analysis

Discriminant validity was assessed according to the Fornell-Lacker criterion (Fornell and Cha, 1994). According to this criterion, if the square root of the AVE of each latent variable is greater than the correlation coefficients between that latent variable and other latent variables in the measurement model, then the model satisfies the discriminant validity criterion (Hair et al., 2006).

The discriminant validity was assessed using Fornell and Larcker (1981) by comparing the square root of each AVE in the diagonal with the correlation coefficients (off-diagonal) for each construct in the relevant rows and columns. For the self-efficacy—EI construct and the self-efficacy—learning motivations construct, there are little disputes. However, the difference is too small, each with 0.053 and 0.028, respectively, and can be ignored (Rahim and Magner, 1995; Hamid et al., 2017). Overall, discriminant validity can be accepted for this measurement model.

Table 2 shows that the mean values of self-efficacy, EI, learning motivation and academic achievement were 3.631, 3.604, 3.571, and 80.968, respectively. The mean values of self-efficacy, EI and learning motivations were between 3.5 and 4. The correlations of the variables all reached significance (*p* < 0.001). These correlations led to further verification of the overall model in this study.

**TABLE 2:** The AVE and correlation coefficients of all variables (*N* = 404).

	Self-efficacy	EI	Learning motivations	Academic achievement
Self-efficacy	0.676^a^			
EI	0.729***	0.781^a^		
Learning motivations	0.704***	0.705***	0.761^a^	
Academic achievement	0.259***	0.275***	0.210***	-
Mean	3.631	3.604	3.571	80.968
Standard deviations	0.535	0.535	0.604	11.819

***p < 0.001.

*^a^Square root of AVE (average variance extracted).*

### Path Analysis of the Overall Model

Firstly, a goodness of fit test of the overall model was performed. Secondly, the path analysis of the overall model related to EI, learning motivation, self-efficacy and academic achievement of the university students in Shanghai was implemented. As for the scale’s goodness of fit test, the three aspects suggested by Hair et al. (2006) were taken as a reference, namely “measures of absolute fit,” “incremental fit measures,” and “parsimonious fit measures.” The results were as follows. In terms of measures of absolute fit: χ^2^ = 1,509.224, df = 621, χ^2^/df = 2.430, which was close to the requirement of χ2/df < 3. RMSEA was 0.060, which was acceptable as it was lower than 0.08. The results reveal that GFI was 0.826 and AGFI was 0.803, which met the criteria of 0.80 (Doll et al., 1994). SRMR was 0.0747, which met the criteria of less than 0.08 (Hu and Bentler, 1999). As for incremental fit measures, the CFI was 0.909, IFI was 0.910 and NNFI was 0.856, which met or was close to the criteria of 0.09. For parsimonious fit measures, the PNFI, PGFI, and PCFI were 0.798, 0.730, and 0.848, respectively, exceeding the criteria of 0.50 (Ullman, 2001). This indicates the overall model exhibited goodness of fit.

**FIGURE 1:**
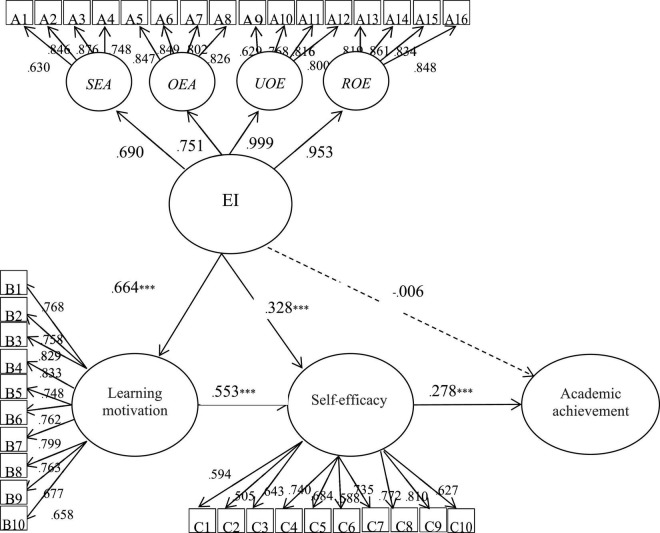
SEM path analysis. SEA, self-emotion appraisal; OEA, others’ emotional appraisal; UOE, use of emotion; ROE, regulation of emotion. ***p < 0.001.

As shown in Figure 1 and Table 3, the path coefficients of the students’ EI related to their learning motivation and self-efficacy were 0.664 (*p* < 0.05) and 0.328 (*p* < 0.05), respectively, which indicates that the students’ EI had a significant positive effect on their learning motivation and self-efficacy.

**TABLE 3:** Bootstrap SEM analysis of total, direct, and indirect effects.

Effect	Estimate	*p*	Confidence Interval
*Direct effect*			
EI→Learning Motivation	0.664	<0.05	[0.582, 0.742]
EI→Self-efficacy	0.328		[0.209, 0.452]
EI→Academic Achievement	–0.006	>0.05	[-0.149, 0.131]
Self-efficacy→Academic Achievement	0.278	<0.05	[0.132, 0.420]
Learning Motivation→Self-efficacy	0.553	<0.05	[0.423, 0.677]
*Indirect effect*			
EI→Self-efficacy	0.368	<0.05	[0.281, 0.474]
Learning Motivation→Academic Achievement	0.154	<0.05	[0.075, 0.241]
EI→Academic Achievement	0.193	<0.05	[0.093, 0.303]
*Total effect*			
EI→Academic Achievement	0.187	<0.05	[0.093, 0.278]

However, the path coefficient of the students’ EI related to their academic achievement was -0.006 (*p* > 0.05) (Table 3 and Figure 1), which shows that the students’ EI did not have a positive effect on their academic achievement. This demonstrates that the higher the student’s EI, the higher their learning motivation (Dubey, 2012; Henter, 2014) and self-efficacy (Hamdy et al., 2014; Gharetepeh et al., 2015; Gurbuz et al., 2016). However, the levels of the students’ EI did not have an effect on their academic achievement, which does not correspond with various research studies and is worth noting (Shamradloo, 2004; Márquez et al., 2006). Therefore, H_2_ and H_4_ are valid but H_1_ is invalid.

Moreover, the mediation model was tested by using the bootstrapping method proposed by Shrout and Bolger (2002). This model was used to test the accuracy of the estimated value of the mediation effect. The procedure involves resampling which results in the mean value and the 95% confidence interval of the mediation effect (Preacher and Hayes, 2008). If the 95% confidence interval of the mediation effect does not include 0, it indicates that the mediation effect reaches the significance level of *p* < 0.05 (Shrout and Bolger, 2002).

The indirect effect of the students’ learning motivation on their EI and self-efficacy was 0.368 (0.664 × 0.553), while the confidence interval [0.281, 0.474] did not include 0 and reached a significant effect (*p* < 0.05), which indicates that learning motivation carried a mediation effect. In other words, the students’ self-efficacy could be increased by their EI through their learning motivation. Furthermore, the indirect effect of the students’ self-efficacy on their learning motivation and academic achievement was 0.154 (0.553 × 0.278), while the confidence interval [0.075, 0.241] did not include 0, which shows that self-efficacy carried a mediation effect. In other words, the students’ academic achievement could be improved by their learning motivation through their self-efficacy. Therefore, H_4_ is valid.

The total indirect effect of learning motivation and self-efficacy between EI and academic achievement was 0.193 (0.664 × 0.553 × 0.278 + 0.328 × 0.278), while the confidence interval [0.093, 0.303] did not include 0, and the path coefficients were positive, as shown in Table 3 and Figure 1. This shows that the students’ EI had an indirect effect on their learning achievement through self-efficacy. Furthermore, the students’ academic achievement could be enhanced by their EI through the process of their learning motivation and self-efficacy. Therefore, H_5_ is valid.

However, the direct effect of EI on academic achievement was -0.006, while the confidence interval [-0.149, 0.131] included 0, and the total effect was 0.187, while the confidence interval [0.093, 0.287] did not include 0. This indicates that the students’ learning motivation and self-efficacy had a total mediation effect between their EI and academic achievement (Table 3 and Figure 1). As a consequence, through the model verification, the EI of the students in Shanghai, who participated in online English courses, could improve their academic achievement through self-efficacy. Additionally, we found that the relation between emotional intelligence and academic achievement was sequentially mediated by learning motivation and self-efficacy.

## Discussion

The results indicated that the correlation between the EI of the university students in Shanghai and their academic achievement did not reach a significant effect in terms of statistics, which is different from this study’s hypothesis. Humphrey-Murto et al. (2014) also suggest that it appears EI cannot reliably forecast students’ future academic performance, and Zahed-Babelan and Moenikia (2010) found that EI in interpersonal relationships has a negative influence on student’s academic performance when engaged in distance learning. Independent learning is the major element of distance learning, as teachers and students are apart from one another. Consequently, students must be highly engaged in their studies (Zahed-Babelan and Moenikia, 2010). Students who successfully accomplish their studies barely require their teachers’ supervision or encouragement (Gros and López, 2016). In this research study, the students’ EI was measured by self-reporting tools, and their academic achievement was assessed by their scores of the final examination. Nonetheless, several researchers used abilities tests to assess EI and utilized GPA to measure academic achievement (Márquez et al., 2006; Berenson et al., 2008). Moreover, there may be other variables involved in academic achievement such as learning motivation (Ruchi, 2012; Henter, 2014) and self-efficacy (Doménech-Betoret et al., 2017; Udayar et al., 2020), which have been proven to be greatly connected with EI.

In this research, the students’ EI had a positive effect on their learning motivation, which was consistent with Dubey’s (2012) and Henter’s (2014) work. Additionally, the students’ EI positively affected their self-efficacy, which was compatible with a substantial body of research (Gharetepeh et al., 2015; Gurbuz et al., 2016; Ngui and Lay, 2020). These aforementioned studies were involved with physical classes. However, this investigation was based on online lessons. The results suggests that students’ EI assists in improving their learning motivation and self-efficacy. In other words, students with higher EI tend to have higher learning motivation and self-efficacy.

Mediation analysis indicated that the relation between emotional intelligence and academic achievement was sequentially mediated by learning motivation and self-efficacy. This study is based on Social Cognitive Theory and the social cognitive EV-M. In SCT, EI influences one’s self-efficacy and work outcomes (Bandura, 1997; Gundlach et al., 2003). The social cognitive EV-M combines learning motivation, self-efficacy and academic achievement (Doménech-Betoret et al., 2017). When the students were participating in the online courses, their self-efficacy had the mediation effect between their EI and academic achievement, which corresponds with the authors’ research (Udayar et al., 2020) and SCT (Bandura, 1997; Gundlach et al., 2003). Furthermore, the relation between emotional intelligence and academic achievement was sequentially mediated by learning motivation and self-efficacy. This shows despite the fact that students can not interact with their classmates and teachers face to face while involved in online English classes, they can still experience others’ emotions in the process of learning and produce their own emotions based on their understanding of the course, which in turn leads to appropriate reactions (Choudary, 2010; Alhebaishi, 2019) and stimulates learning motivation (Dubey, 2012). Additionally, students with high EI can obtain a higher degree of belief in self-efficacy by managing their own emotions (Bandura, 1997; Gundlach et al., 2003). When students are motivated to learn, they become energized and engaged with their English courses (Schunk and Meece, 2005; Jenkins and Demaray, 2015) and therefore their results improve (Doménech-Betoret et al., 2017; Udayar et al., 2020).

## Conclusion

Due to the impact of the COVID-19 pandemic, there have been a substantial number of schools running online courses. In this study, the EI of Chinese students, who took part in the online English lessons, did not influence their academic achievement.

Students’ EI does not directly affect their academic achievement; however, it directly and positively impacts their learning motivation and self-efficacy. Students, who have higher EI, tend to have higher learning motivation and can feel others’s emotions during online courses, which affects their self-efficacy and indirectly influences their academic achievement. As a consequence, it is still critical for them to properly manage and develop their EI. Schools, which implement online teaching, also need to pay attention to enhancing the development of students’ EI by arranging appropriate online lessons.

Teachers should attach importance to, and advance, students’ learning motivation and self-efficacy when utilizing online courses, since their EI can improve their English academic achievement through their learning motivation and self-efficacy. Thus, learning motivation and self-efficacy play a key role between EI and academic achievement. Researchers could include the concepts of learning motivation and self-efficacy when carrying out future studies on EI and academic achievement. There are still numerous schools conducting online teaching in the world due to the influence of the COVID-19 pandemic. Although the research subjects were specific Chinese students in Shanghai, the conclusion and recommendations of this study can still be a reference to other schools running online courses. These findings are beneficial for the exploration of the complex relation between emotional intelligence and academic achievement.

## Data Availability Statement

The raw data supporting the conclusions of this article will be made available by the authors, without undue reservation.

## Ethics Statement

The studies involving human participants were reviewed and approved by Ethical Committee of Dhurakij Pundit University. The patients/participants provided their written informed consent to participate in this study.

## Author Contributions

Y-CC: responsible for the conceptualization, investigation, methodology, and writing analyzing data for this manuscript. Y-TT: responsible for the suggesting revision to the concept and writing style of the manuscript. Both authors have read and agreed to the published version of the manuscript.

## Conflict of Interest

The authors declare that the research was conducted in the absence of any commercial or financial relationships that could be construed as a potential conflict of interest.

## Publisher’s Note

All claims expressed in this article are solely those of the authors and do not necessarily represent those of their affiliated organizations, or those of the publisher, the editors and the reviewers. Any product that may be evaluated in this article, or claim that may be made by its manufacturer, is not guaranteed or endorsed by the publisher.
